# Assessing the socioeconomic and environmental determinants of flood vulnerability in India: a panel data approach

**DOI:** 10.1038/s41598-025-09442-9

**Published:** 2025-07-30

**Authors:** Kannan Thomas Felix, M. Balasubramanian, Padma Lakshmi Govindarajan, B. Kesav

**Affiliations:** 1 Agriculture Development and Rural Transformation Centre, Institute for Social and Economic Change, Bengaluru, Karnataka 560 072 India; 2 Centre for Ecological Economics and Natural Resources, Institute for Social and Economic Change, Bengaluru, Karnataka 560 072 India; 3 Department of Agricultural Extension and Economics, VIT School of Agricultural Innovations and Advanced Learning (VAIAL), Vellore Institute of Technology, Vellore, Tamil Nadu 63201 India; 4 VIT School of Agricultural Innovations and Advanced Learning (VAIAL), Vellore Institute of Technology, Vellore, Tamil Nadu 632014 India

**Keywords:** Flood vulnerability, Panel data modelling, Returns to scale, Disaster mitigation, Environmental sciences, Natural hazards

## Abstract

This study investigates the socioeconomic and environmental determinants of flood vulnerability in Indian states using a panel data approach. Drawing on state-level flood damage data from 1953 to 2020, sourced from the Ministry of Jal Shakti and affiliated agencies, this study employs a robust econometric framework to identify the key drivers of flood-related damage. The dataset included 16 variables, and rigorous preprocessing steps, such as data cleaning, interpolation, stationarity testing, and multicollinearity checks, to ensure model reliability. Endogeneity was tested using IV regression, and the absence of such issues allowed for the application of Fixed Effects (FE) and Random Effects (RE) models. The Hausman test favored the RE model, which was further validated through diagnostic and robustness tests. The findings reveal that capital expenditure, government spending on natural calamities, and mangrove coverage significantly reduce flood damage, highlighting the importance of both fiscal and ecological interventions. Conversely, population affected, damage to houses, and damage to public utilities were positively associated with flood damage, indicating the need for targeted infrastructure and community resilience strategies. This study offers actionable insights for policymakers, emphasizing the integration of environmental conservation with strategic public investment to enhance flood resilience in India.

## Introduction

Flood damage in India poses a significant economic challenge, warranting a thorough econometric analysis to comprehend the extent and implications of these events. India is prone to floods owing to its diverse topography, climatic variations, and high population density in flood-prone regions^1^. The economic impact of floods is multifaceted, affecting agriculture, infrastructure, income, employment, health, children’s education, and overall productivity^2–7^. Agriculture, a cornerstone of the Indian economy, is particularly vulnerable, with floods causing crop damage, soil erosion, and livestock loss^8,9^.

The main causes of floods are various anthropogenic and other factors, such as land use change^10,11^, unplanned urbanization^12,13^, and deforestation^14–17^. Several flood-related studies have been conducted at the local, regional, and global levels. For example, Kurtilla^18^ pointed out flood damages and insurance in the United States of America; Bremond et al.^19^ discussed the impact of floods on the agricultural sector; and Tanoue et al.^20^ examined both the direct and indirect economic impacts of flood damages in Thailand. Davies^21^ assessed the social costs related to floods, and Dottori et al.^22^ estimated human loss and economic impacts due to river floods at the global level. However, India is one of the countries that has a negative impact on climate-related damage. For example, Singh and Kumar^23^ assessed the economic impacts of floods in India and estimated an economic loss of US$ 16 billion from 1978 to 2006. Patri et al.,^24^ examined flood damages from 1981 to 2019 and its impact on state gross domestic product at the state level. Dottori et al.^22^ also estimated the economic and welfare loss due to flood related calamities and found that the losses were very high in India compared to other Asian counties. Parida^25^ estimated the economic impact of floods in 19 Indian states and found a positive correlation between per capita income and flood related fatalities due to government spending on flood control measures and strategies in India. Floods have negatively affected the manufacturing and services sectors due to human loss and health impacts in many Indian states^26^. Economic estimates of flood damages accounted for Rs 66,550 per hectare in Chennai^27^. The direct and indirect economic impacts of flood damages in Mumbai were estimated to be US$ 2 billion and 500 fatalities, respectively^28^.

While reviewing the methodologies, we found a lack of empirical econometric analysis on flood damages in India. For instance, Gupta et al.^29^ examined the economic impacts of flood damages in three Indian states, including Uttar Pradesh, Bihar, and West Bengal, during 1971 – 1996. This study used a regression model, and the results revealed that a lack of precautionary measures and inadequate flood protection have increased the damages in the study states. Parida and Prasad^30^ examined the economic impacts of flood damage in selected Indian states. He used a Tobit regression model between per capita income and flood impacts. Other studies include Patri et al.^24^, who used Zero-Inflated Negative Binomial Regression (ZINB) to find the association between economic growth and flood damages in selected Indian states from 1981 to 2019. Panwar and Sen^26^ used an augmented panel vector auto-regression econometric model to study flood damage and its economic impacts in selected states of India. Amarasinghe et al.^31^ used fixed effects panel regression model to study the association between flood, drought and Gross State Domestic Product (GSDP) and Human Development in India for the period from 2001 to 2015.

The econometric analysis of flood damage necessitates a comprehensive understanding of the factors contributing to economic losses^32^. Rainfall patterns, river basin characteristics, land use changes, and urbanization are key variables influencing the frequency and intensity of floods and their economic repercussions^33^. Using advanced econometric models, researchers have attempted to quantify the economic losses associated with these variables. For instance, studies have employed panel data techniques to analyze the impact of annual precipitation variations on agricultural output, revealing that crop insurance may stabilize revenues and protect farmers from exposure to increasing weather-related risks^34^. The infrastructure sector bears a substantial burden of flood damage, with roads, bridges, and buildings often facing destruction or impairment. An econometric assessment of the economic consequences of such damage involves modelling the relationship between flood-induced infrastructure disruptions and their subsequent effects on industries and regional economies. This requires a nuanced analysis that considers factors such as accessibility, transportation costs, and the resilience of infrastructure systems. Furthermore, the socio-economic dimension of flood damage cannot be overlooked. Vulnerable populations, including those living in informal settlements and low-lying areas, are disproportionately affected by flooding. An econometric examination of social vulnerabilities and their correlation with flood impacts necessitated the integration of demographic data, income levels and access to resources^35^. Such analyses can provide valuable insights into the distributional aspects of flood damage and targeted policy interventions. Government policies and institutional frameworks also play crucial roles in mitigating flood damage. An econometric analysis can shed light on the effectiveness of existing policies, such as disaster management strategies and early warning systems, in reducing the economic losses associated with floods^36^. This involves assessing the impact of policy variables on the severity of flood damage and identifying areas for improvement. The econometric analysis of flood damage in India is a multifaceted endeavor that requires a holistic understanding of the various factors contributing to economic losses. By employing advanced econometric models, researchers can unravel the intricate relationships between climatic, geographic, economic and policy variables. Against this background, the present study aims to assess the key socioeconomic and environmental determinants influencing flood vulnerability across Indian states using a panel data approach.

While several studies have examined flood damage in India, there is a lack of comprehensive empirical econometric analyses that integrate socioeconomic and environmental factors. Most existing studies focus on specific states or regions, use limited time periods, or employ narrow methods. The proposed study addresses this gap by utilizing a panel data approach to analyze flood vulnerability across India, incorporating a wider range of determinants. This approach would allow for a more nuanced understanding of the complex interplay between socioeconomic factors (such as population density and income levels) and environmental variables (such as rainfall patterns, land use changes, and river basin characteristics) in determining flood vulnerability. Additionally, this study contributes to the limited body of research on the effectiveness of government policies and institutional frameworks in mitigating flood damage, providing valuable insights for evidence-based policymaking and sustainable development in flood-prone areas.

The principal contribution of this study is that it addresses a significant research gap by conducting a comprehensive empirical econometric analysis of flood vulnerability in India. It uses a panel data approach to examine flood vulnerability across the country, integrating a broad range of socioeconomic and environmental determinants. This research provides a nuanced understanding of the complex interactions between these factors and contributes to the limited research on the effectiveness of government policies and institutional frameworks in mitigating flood damage. By offering valuable insights, this study aims to support evidence-based policymaking.

The paper begins with an introduction that outlines the study’s principal contributions and establishes the need for research. The Literature Review section covers various aspects related to floods, including their economic impact, vulnerability and resilience, cost–benefit analysis, spatial distribution of damage, and policy interventions. The Methodology section describes the analytical approaches used, such as fixed effects and random effects models, as well as statistical tests such as the Breusch-Pagan Lagrange Multiplier test and Hausman test. The Results and Discussion section presents the findings and their interpretations. The paper concludes with a Conclusion and Policy Implications section, summarizing the key findings and their relevance to policymaking. Additional sections include Data Availability, Ethical Approval, Informed Consent, and Funding, which provide transparency regarding the research process and resources. The paper ends with a References section, listing the sources cited throughout the study.

## Review of literature

### Economic impact of floods

The economic impact of floods in India is profound, affecting various sectors and posing significant challenges to the country’s growth and development. Direct economic consequences primarily include extensive damage to infrastructure, such as roads, bridges, and buildings. Reconstructing and repairing these infrastructures necessitate substantial financial resources, which can strain public and private budgets^29^. Floods often disrupt transportation networks, impeding the movement of goods and people, thereby affecting commerce and daily activities^37^. Agriculture, a vital sector of India’s economy, is particularly vulnerable to flooding. Floodwaters can destroy crops, erode fertile soil, and disrupt the agricultural supply chain, leading to significant losses for farmers and increased food prices for consumers^31^. These agricultural damages not only reduce immediate harvest yields but can also lead to long-term soil degradation, thereby affecting future agricultural productivity^38^. In urban areas, floods can cause extensive damage to businesses, leading to a halt in economic activities and loss of income. The destruction of commercial properties, inventories, and critical infrastructure results in substantial financial losses for businesses^39^. Indirect economic consequences of floods include a slowdown in economic growth. Frequent flooding events can deter investment because businesses and investors perceive higher risks in flood-prone areas. This aversion to investment can stymie economic development and exacerbate poverty in the affected regions^30^. Additionally, the financial burden of flood recovery can divert public funds from other critical areas, such as education, healthcare, and infrastructure development, further hampering long-term growth^23^. Floods have significant implications for financial systems. The increase in insurance claims and the necessity for emergency funding can strain financial institutions and public finances. This financial stress can inhibit financial development and reduce economic resilience, making it more challenging for regions to recover from future disasters^40^. Moreover, the economic impact of floods is not uniformly distributed across all states, with some regions experiencing more severe effects owing to varying levels of preparedness and resilience^24^. The cumulative impact of floods on India’s GDP was substantial. Flood-related damage can lead to a significant reduction in economic output, affecting both immediate economic performance and long-term growth prospects. Studies have shown that recurrent flooding can lead to a persistent slowdown in economic activities, highlighting the need for effective flood management and mitigation strategies to minimize these adverse effects^26^. Effective flood management, including investment in flood defenses and early warning systems, can mitigate some of the direct damage and reduce the overall economic impact of floods^29^. Recent machine learning approaches for flood prediction and agriculture have shown significant advancements in the utilization of recurrent neural networks (RNNs) and optimization techniques. In the field of crop disease prediction, a novel framework based on ensemble learning techniques and spatio-temporal recurrent neural networks (STRNN) has been proposed, which outperforms baseline models in predicting crop disease severity^41^. This approach considers both temporal and spatial dependencies, thereby offering improved accuracy for agricultural emergency management. For crop yield forecasting, weight-agnostic neural networks (WANN) optimized by modified metaheuristics have demonstrated promising results. These models achieved a mean absolute error of 0.012 and an R-squared score of 0.89, indicating strong predictive performance^42^. Additionally, deep learning-based RNN models, particularly Long Short-Term Memory (LSTM) networks, have shown superior performance in predicting wheat crop yield compared to traditional machine learning models^43^. Interestingly, although RNNs and LSTMs have proven effective in various agricultural applications, some studies have found that simple baselines can achieve state-of-the-art performance in certain tasks, such as human motion prediction^44^. This highlights the importance of carefully evaluating the model complexity and performance across different domains. In conclusion, the integration of advanced neural network architectures, such as RNNs and LSTMs, combined with optimization techniques such as metaheuristics, represents the current state-of-the-art in flood prediction and agricultural applications. These approaches offer improved accuracy and efficiency in predicting crop yields, disease severity, and other time-dependent agricultural phenomena, while also providing valuable insights for decision-making and resource management in the agricultural sector.

In summary, floods in India have far-reaching economic impacts, encompassing both direct damage to infrastructure and agriculture and indirect effects on economic growth and financial stability. Addressing these challenges requires comprehensive flood management strategies and investments in resilient infrastructure to safeguard the economy from future flood-related disruptions^26,40^.

### Vulnerability and resilience

Flood vulnerability in India is shaped by a complex interplay of socioeconomic, demographic, and infrastructural factors. Poverty is a critical determinant, as economically disadvantaged populations often reside in flood-prone areas because of lower land costs and limited access to safer locations. These communities typically lack the financial resources to invest in flood defences or recover swiftly from flood-related damages, thereby exacerbating their vulnerability^45,46^. High population density, particularly in urban areas, further compounds vulnerability by placing a greater strain on existing infrastructure and services. This increased pressure can lead to inadequacies in drainage systems and exacerbate the impacts of flooding, as observed in cities such as Delhi and Chennai^47,48^. Inadequate infrastructure, such as poor-quality housing and insufficient drainage systems, significantly increases flood risk. In rural areas, the lack of robust infrastructure means that homes and livelihoods are easily disrupted by flood events, leading to prolonged recovery periods and economic instability^49^. Furthermore, regions with poor early warning systems and limited emergency response capabilities are less able to mitigate the immediate impacts of floods, resulting in higher casualties and greater property damage^50^. Several strategies have been identified to enhance resilience against flood damage. Strengthening infrastructure is paramount, including upgrading drainage systems, constructing flood barriers, and improving housing quality in vulnerable areas. Investing in resilient infrastructure helps reduce the physical impacts of flooding and speeds up recovery times^51^. In addition, improving early warning systems and emergency response mechanisms can significantly mitigate the adverse effects of flooding. Timely and accurate dissemination of flood warnings allows communities to prepare and evacuate if necessary, thereby reducing casualties and property damage^52^. Community-based approaches to resilience are essential. Empowering local communities through education and capacity-building initiatives enhances their ability to respond to and recover from flood events. This includes training in disaster preparedness and response and fostering local leadership and cooperation in disaster management efforts^48^. Integrating traditional knowledge with modern technology can enhance resilience, as local practices and experiences often provide valuable insights into effective flood management strategies^53^. Additionally, addressing the root causes of vulnerability, such as poverty and inequity, is crucial for achieving long-term resilience. Economic development initiatives that provide stable livelihoods and reduce poverty can significantly enhance communities’ capacity to withstand and recover from floods. This includes promoting sustainable agricultural practices, diversifying income sources, and ensuring access to financial services, such as insurance^46,49^. Remote sensing and GIS technologies play critical roles in flood vulnerability mapping and risk assessment. These tools can identify high-risk areas and inform targeted interventions, allowing for more efficient resource allocation and better planning of flood mitigation measure^54^. By combining technological advancements with community engagement and robust infrastructure development, a comprehensive approach to building resilience against flood damage can be achieved^51^. In summary, reducing flood vulnerability and enhancing resilience in India requires a multifaceted approach that addresses socioeconomic disparities, strengthens infrastructure, improves early warning systems, and empowers communities. By integrating these strategies, India can better protect its population and minimize the economic and social impacts of flood events^50,55^.

### Cost–benefit analysis

Conducting a cost–benefit analysis (CBA) for flood prevention measures in India involves comparing the financial outlays required for implementing these measures with the economic benefits realized through the reduction of flood damages and associated losses. Embankments, early warning systems, and other flood mitigation strategies require significant initial investments, but their potential to avert substantial economic and social costs makes them valuable. The construction of embankments, for instance, has been a traditional method of flood protection in regions such as the Brahmani–Baitarani River Basin. Although the upfront costs of building and maintaining these structures are high, studies have shown that they effectively reduce the frequency and severity of flood events, thereby protecting agricultural land, infrastructure, and human settlements from extensive damage^56^. The long-term benefits of embankments include safeguarding livelihoods and preventing displacement, which can lead to considerable economic savings and stability for the affected communities^57^. Early warning systems represent another critical investment in flood-risk management. The costs associated with developing and maintaining these systems include technological infrastructure, training personnel, and continuous updates to ensure their accuracy and reliability. However, the benefits of early warning systems are significant. By providing timely alerts, these systems enable communities to evacuate, secure properties, and take precautionary measures, thereby significantly reducing human casualties and economic losses^58^. In India, the implementation of early warning systems has proven effective in minimizing the impacts of flood events, thereby demonstrating their value in disaster risk reduction^59^. A broader cost–benefit perspective also considers the indirect benefits of flood prevention measures. Investments in flood-proofing low-income houses, for example, not only protect assets but also enhance the overall resilience of vulnerable populations to climate change. Such interventions can prevent the cascading effects of flooding on poverty and socioeconomic development, creating a more stable and prosperous community^60^. Moreover, these measures contribute to long-term economic efficiency by reducing the need for repeated disaster relief and reconstruction efforts^61^. Probabilistic cost–benefit analysis (CBA), which incorporates the likelihood of various flood scenarios and their potential impacts, provides a more nuanced understanding of the economic efficiency of flood prevention measures. This approach helps policymakers prioritize interventions that offer the greatest return on investment under uncertain future conditions^62^. For instance, investing in a combination of structural measures (such as embankments) and non-structural measures (such as early warning systems and community-based disaster preparedness) can maximize benefits by addressing different aspects of flood risk^63^. Overall, the evidence supports that while the costs of flood prevention measures are substantial, the benefits in terms of reduced damage, economic stability, and enhanced resilience are significant. Effective flood risk management through cost–benefit analysis enables informed decision-making that balances immediate expenditures with long-term gains, thereby ensuring sustainable economic development and disaster resilience^45,46^. Such analyses underscore the importance of proactive investment in disaster risk reduction to mitigate the socioeconomic impacts of floods in India^61^.

### Spatial distribution of damages

The spatial distribution of flood damage across India exhibits significant regional variations, influenced by factors such as land use, urbanization, and climate change. Urbanization has particularly exacerbated flood risks in densely populated cities, such as Mumbai and Chennai, where extensive impervious surfaces hinder natural drainage, leading to increased flood susceptibility. In Mumbai, the Poisar River Basin has experienced intensified flood hazards owing to land use-land cover changes, which have reduced the basin’s capacity to absorb and channel water effectively^64^. Similarly, urban sprawl in Chennai has heightened future flood risks by altering natural water flow patterns and reducing green spaces that can mitigate flooding^65^. Regional disparities in flood damage are also shaped by socioeconomic factors and local governance. Coastal cities, such as Mumbai, face unique challenges owing to their geographical location and high population density. Studies indicate that climate change will exacerbate these challenges by increasing the frequency and intensity of heavy rainfall events, leading to more severe and frequent floods^28,66^. The economic and social impacts of such events are profound, often disproportionately affecting the urban poor who live in vulnerable areas with inadequate infrastructure^67^. In rural and semi-urban areas, the impact of land use changes on flood risk is similarly significant. The Oshiwara River Basin in Mumbai is a prime example of urbanization drastically altering land cover, resulting in increased flood risk due to the loss of natural floodplains^13^. In these regions, agricultural lands and rural settlements are often less resilient to flood impacts, leading to extensive economic damage and long recovery periods. Climate change acts as a multiplier of the existing vulnerabilities. Future flood hazards are projected to become more severe because of changing precipitation patterns and rising sea levels. Mapping studies have highlighted that both climatic factors and anthropogenic land use changes will significantly enhance the susceptibility of Indian regions to flooding in the coming decades^68,69^. This increasing flood risk necessitates adaptive strategies that consider both current and future scenarios to effectively mitigate the damage. Adaptation measures vary by region and include both structural and nonstructural approaches. Coastal cities benefit from enhanced flood defenses, improved drainage infrastructure, and integration of green infrastructure to manage stormwater. In rural areas, the restoration of natural floodplains and the implementation of sustainable land management practices can reduce flood risks. Effective governance and community-based approaches are crucial for tailoring these measures to local conditions and ensuring their successful implementation^70^. In summary, the spatial distribution of flood damage in India is a complex interplay of urbanization, land use changes, and climate change. Urban areas, such as Mumbai and Chennai, face heightened flood risks due to dense development and inadequate drainage, whereas rural areas are impacted by altered land use and limited adaptive capacity. Addressing these challenges requires a multifaceted approach that includes robust infrastructure, sustainable land management, and proactive climate-adaptation strategies^66,71^.

### Policy interventions

Evaluating existing policies for flood management, disaster response, and risk reduction in India reveals a multifaceted approach that integrates climate adaptation, social protection, and community-based strategies. Several policies have been implemented with varying degrees of success, highlighting both their strengths and areas for improvement. One notable policy intervention is the development of South Asia’s first Heat-Health Action Plan in Ahmedabad, Gujarat, which serves as a model for integrating climate adaptation into public-health planning. This plan includes early warning systems, public awareness campaigns, and capacity building for healthcare professionals, demonstrating a comprehensive approach to managing climate-related health risks^72^. The principles and strategies of this plan can be adapted for flood management by incorporating early warning systems and community engagement to enhance preparedness and response efforts. In the agricultural sector, climate change adaptation and disaster risk reduction (DRR) policies are essential for protecting rural livelihoods and ensuring food security. Research suggests that integrating social protection measures, such as insurance schemes and cash transfers, with climate-adaptation strategies can significantly enhance the resilience of agricultural communities. These complementary roles support rural growth by mitigating the adverse impacts of floods and other climate-related disasters^73^. Watershed management and forest conservation play crucial roles in mitigating floods. Evidence-based approaches to integrated flood management emphasize the importance of maintaining healthy forest ecosystems to regulate water flow and reduce flood risk. Policies that promote afforestation, sustainable land use practices, and the restoration of degraded watersheds can effectively mitigate flood hazards^74^. Such policies should be strengthened and expanded to ensure long-term resilience to flooding. The Sendai Framework for Disaster Risk Reduction (2015–2030) provides a global blueprint for reducing disaster risks, including floods. Assessing contributions to this framework through various climate adaptation and DRR projects across the Asia–Pacific highlights the importance of multi-sectoral approaches and the integration of DRR into national and local development plans^75^. Indian cities, such as Mumbai and Chennai, are increasingly incorporating climate change adaptation measures into urban planning to address the challenges posed by urbanization and climate variability^76^. Decentralization and evidence-based decision-making are critical components of effective disaster risk management. By empowering local governments and utilizing data observatories to inform policy decisions, India can improve its disaster resilience further. The use of indices and data-driven approaches allows targeted interventions that address specific vulnerabilities and enhance the overall effectiveness of DRR strategies^77^. Despite these efforts, challenges remain in implementing early warning systems and community engagement. Effective early warning systems require not only technological advancements but also societal readiness, including public awareness and trust in the system^78^. Strengthening community-based disaster preparedness and response mechanisms is essential to ensure timely and effective actions during flood events. Furthermore, systematic reviews of farmers’ adaptation strategies reveal the need for policies that support sustainable agricultural practices and provide financial and technical assistance to farmers. These measures can enhance adaptive capacity and reduce vulnerability to flooding^79^. In conclusion, although existing policies in India demonstrate a comprehensive approach to flood management and disaster risk reduction, there is a need for continuous improvement and adaptation. Recommendations for enhancing flood resilience include strengthening early warning systems, promoting sustainable land and water management practices, integrating social protection with climate adaptation, and empowering local governments through decentralization and data-driven decision making^80,81^. These evidence-based strategies can significantly reduce the socioeconomic impacts of floods and enhance the resilience of vulnerable communities.

## Methodology

For the present study, we utilized state-wise flood damage statistics from 1953 to 2020, provided by the Ministry of Jal Shakti, Deptt. of Water Resources, RD & GR, Central Water Commission, and Flood Forecasting Monitoring Directorate (http://www.indiaenvironmentportal.org.in/content/472302/flood-damage-statistics-statewise-and-for-the-country-as-a-whole-during-1953-to-2020/), released in 2022. The variables used in the model are presented in Table 1.

**Table 1:** Variables description.

S.No	Dependent variable			Description	Data Source
1	$TD$			Value of Total damages crops, houses & public utilities n Rs. Crore	State wise flood damage, Indian environmental portal, Government of India
	Independent variables				
2	Exogenous Variables	Environmental variable	$ARF$	Actual/Observed Rainfall (in mm)	Observed rainfall data (1901–2025) is sourced from various IMD publications under the Ministry of Earth Sciences, Government of India
3		Socioeconomic variable	$PCNSDP$	Per Capita NSDP (Constant Prices)	Back series data were used
4		Environmental variable	$FA$	Forest Area (sq. km)	Forest area data (1991 to 2021) is sourced from the State of Forest Report-Various Issues, Forest Survey of India, Ministry of Environment and Forest, Government of India
5		Socioeconomic variable	$TP$	Total Population (numbers)	Census data, Government of India
6		Environmental Variable	$AA$	Area Affected (m.ha.)	State wise flood damage, Indian environmental portal, Government of India
7		Environmental variable	$DTCA$	Damage to Crops Area (m.ha.)	State wise flood damage, Indian environmental portal, Government of India
8		Socioeconomic variable	$CL$	Cattle Lost (Nos)	State wise flood damage, Indian environmental portal, Government of India
9		Socioeconomic variable	$HLL$	Human Lives Lost (Nos)	State wise flood damage, Indian environmental portal, Government of India
10	Endogenous variables	Socioeconomic variable	$CE$	Capital Expenditure (Rs Crore)	Sourced from the Comptroller and Auditor General (CAG) of India (www.cag.nic.in)
11		Socioeconomic variable	$RE$	Revenue Expenditure (Rs Crore)	Sourced from the Comptroller and Auditor General (CAG) of India (www.cag.nic.in)
12		Socioeconomic variable	$GENC$	Government Expenditure on Natural Calamities (Rs Lakh)	Expenditure on natural calamities data (1972–73 to 2023–24) is source from State Finances: A Study of Budgets, Reserve Bank of India (RBI)
13		Socioeconomic variable	$PA$	Population Affected (million)	State wise flood damage, Indian environmental portal, Government of India
14		Socioeconomic variable	$DTH$	Damage to Houses (Nos)	State wise flood damage, Indian environmental portal, Government of India
15		Socioeconomic variable	$DTPU$	Damage to Public Utilities (Rs. Crore)	State wise flood damage, Indian environmental portal, Government of India
16		Environmental Variable	$MC$	Mangrove Cover (sq. km)	Mangrove Cover data (1987 to 2021) is sourced from the Environment Statistics—Vol. I, National Statistical Office, Ministry of Statistics & Programme Implementation, Government of India

### Interpolation

Based on an extensive review, this study utilized a panel data approach to examine flood vulnerability determinants across Indian states from 1953 to 2020.

Interpolation was performed for the missing values. When we have two known data points (x_1_,y_1_) and (x_2_, y_2_), the formula for interpolation is

$$y={y}_{1}+\frac{(x-{x}_{1}) ({y}_{2}-{y}_{1})}{{x}_{2}-{x}_{1}}$$

where, x is the year, and y is the variable being interpolated (e.g., td, arf, etc.). The formula fills in the missing values by drawing a straight line between the two nearest known values of y on either side of the missing year.

### Panel unit root test (ADF within fisher test)

For each panel unit i, the ADF regression is as follows:

$${\Delta y}_{it}={\alpha }_{i}+{\beta }_{i}{y}_{i, t-1}+\sum_{j=1}^{p}{\gamma }_{ij}{\Delta y}_{i, t-j}+{\varepsilon }_{it}$$

The Null hypothesis is ${\beta }_{i}=0$, unit root/ non stationary and the Alternate hypothesis is ${\beta }_{i}<0$, stationary.

The fisher test combines p values from individual ADF tests

$${\chi }^{2}=-2\sum_{i=1}^{N}ln \left({p}_{i}\right)$$

where, ${p}_{i}$ is the value from ADF test for unit root I and N is the number of panel units.

If the variable y_it_ is non-stationary, we transform it by taking the first difference, as follows:

$${\Delta y}_{it}={y}_{it}-{y}_{i, t-1}$$

Then we ran unit root test on ${\Delta y}_{it}$

$${{\Delta }^{2}y}_{it}={\alpha }_{i}+{\beta }_{i}{y}_{i, t-1}+\sum_{j=1}^{p}{\gamma }_{ij}{{\Delta }^{2}y}_{i, t-j}+{\varepsilon }_{it}$$

### Multicollinearity checks

To check for multicollinearity, we performed a panel regression followed by the Variance Inflation Factor (VIF) formula. The panel data regression was done using the formula

$${y}_{it}=\alpha +\beta {X}_{it}+{u}_{it}$$

${y}_{it}$ dependent variable for entity i at time t; ${X}_{it}$ vector of independent variables for entity i at time t; $\alpha$ intercept; $\beta$ coefficients; ${u}_{it}$ error term.

After the panel regression, VIF for each predictor ${X}_{it}$ was calculated using

$$VIF=\frac{1}{1-{R}_{j}^{2}}$$

${R}_{j}^{2}$ is the R-squared from regressing ${y}_{it}$ on all other independent variables, A high ${R}_{j}^{2}$ means ${y}_{it}$ is highly collinear with other predictors. A VIF > 10 is often considered a sign of serious multicollinearity issues.

### Endogeneity test

We performed a test for endogeneity using IV regression with multiple endogenous variables to test for endogeneity, instrument strength, and overidentifying restrictions.

Structural Equation (Main Model)

$${Y}_{it}={\beta }_{1}{X}_{1, it}+{\beta }_{2}{X}_{2, it}+\dots +{\beta }_{15}{X}_{15, it}+{\varepsilon }_{it}$$

${Y}_{it}$: Dependent variable for unit i at time t; ${\beta }_{1}{X}_{1, it} to {\beta }_{15}{X}_{15, it}$: Endogenous regressors; ${\varepsilon }_{it}$: Structural error term.

#### First-stage regressions (one for each endogenous variable)

Each endogenous regressor ${X}_{j, it}$ ​ is regressed on a set of instruments ${Z}_{1, it}$​,…,${Z}_{8, it}$​:

$${X}_{j, it}={\pi }_{j0}+{\pi }_{j1}{Z}_{1, it}+\dots +{\pi }_{j8}{Z}_{8, it}+{v}_{j, it}$$

where: ${Z}_{1, it}$​,…,${Z}_{8, it}$​: Instrumental variables (must be relevant and exogenous); ${v}_{j, it}$: First-stage error term.

The Null Hypothesis of the test for endogeneity (Durbin-Wu-Hausman Test) is that all regressors are exogenous, and the alternative hypothesis is that at least one regressor is endogenous.

#### Overidentifying restrictions (Hansen J or Sargan test)

Applicable when the model is over-identified (more instruments than endogenous variables).

$$J=n.{R}_{IV Residual regression}^{2}$$

The null hypothesis is the instruments are valid (uncorrelated with error term); $J\sim {\chi }_{q}^{2}$, where q = number of overidentifying restrictions q = number of overidentifying restrictions.

The methodology involved fixed effects and random effects models and Hausman tests used to determine the most appropriate model since the model does not have an endogeneity issue. The random effects model was ultimately selected, allowing for the analysis of both time-invariant and time-varying factors affecting flood vulnerability.

### Fixed effects model (FE)

The fixed effects model for the present study is presented in Eq. (1). It allows individual-specific effects ${\alpha }_{i}$ to be correlated with the regressors $x$ by including them ${\alpha }_{i}$ as entity-specific intercepts (fixed effects). $+{u}_{it}$ is the error term. As per the Eq. (1), each individual has a unique intercept term while sharing the same slope parameters (Brüderl and Ludwig, 2015).

1 $$\begin{aligned} TD_{{it}} = & \alpha _{i} + \beta _{1} CE_{{1,it}} + \beta _{2} RE_{{2,it}} \\ & + \beta _{3} GENC_{{3,it}} + \beta _{4} PA_{{4,it}} \\ & + \beta _{5} DTH_{{5,it}} + \beta _{6} DTPU_{{6,it}} \\ & + \beta _{7} MC_{{7,it}} + \varepsilon _{{it}} \\ \end{aligned}$$

Where: ${TD}_{it}$: Dependent variable; ${CE}_{1,it}, {RE}_{2,it}, {GENC}_{3,it}, {PA}_{4,it}, {DTH}_{5,it}, {DTPU}_{6,it} and {MC}_{7,it}$: Independent variables; ${\alpha }_{i}$: Entity-specific intercept (captures unobserved heterogeneity) and ${\varepsilon }_{it}$: Idiosyncratic error term. The key assumption is ${\alpha }_{i}$ may be correlated with the regressors (independent variables). It is important to note that, although the variable description section lists 15 independent variables, only 7 are included in the regression equations. This is because the remaining 8 variables are treated as exogenous and serve as strong instrumental variables (IVs) for the 7 endogenous regressors. Therefore, only the endogenous variables are included in the Fixed Effects (FE) and Random Effects (RE) models, while the exogenous variables are used exclusively in the instrumental variable estimation.

### Random effects model (RE)

The Random effects model for the present study is presented in Eq. (2). The RE model assumes that the entity-specific effects are random and uncorrelated with the regressors.

2 $$\begin{aligned} TD_{{it}} = & \alpha + \beta _{1} CE_{{1,it}} \\ & + \beta _{2} RE_{{2,it}} + \beta _{3} GENC_{{3,it}} \\ & + \beta _{4} PA_{{4,it}} + \beta _{5} DTH_{{5,it}} \\ & + \beta _{6} DTPU_{{6,it}} + \beta _{7} MC_{{7,it}} + u_{i} + \varepsilon _{{it}} {\text{ }} \\ \end{aligned}$$

Where: $\alpha$: Common intercept; ${u}_{i}$: Random effect for entity $i,{u}_{i}\sim N \left(0, {\sigma }_{u}^{2}\right).$ Key Assumption is : ${u}_{i}$ ​is uncorrelated with all regressors (independent variables).

### Hausman test

The null hypothesis is that the preferred model is random effects versus alternative fixed effects^83^. It tests whether the unique errors (${\alpha }_{i}$) are correlated with the regressors, the null hypothesis is they are not correlated. The random effects estimator is more efficient; therefore, we need to use it if the Hausman test supports it. The Hausman test statistic can only be calculated for time-varying regressors.

The Hausman test statistic is represented by Eq. (3) as follows:

3 $$H = \left( {\hat{\beta }_{RE} - \hat{\beta }_{FE} } \right){\prime} \left( {V\left( {\hat{\beta }_{RE} } \right) - V\left( {\hat{\beta }_{FE} } \right)} \right)\left( {\hat{\beta }_{RE} - \hat{\beta }_{FE} } \right)$$

### Results and discussion

#### Description of variables

#### Total flood damages (crops, houses & public utilities)

**Fig. 1:**
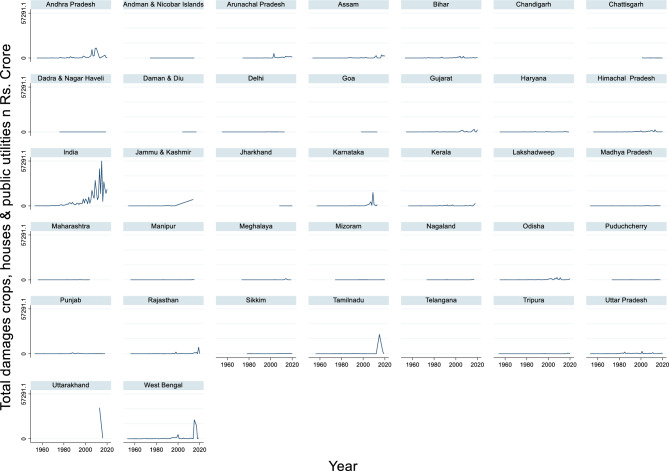
Total flood damage (crops, houses, and public utilities) in Rs. Crore.

From 1953 to 2020, the total flood damage in India due to crops, houses, and public utilities exhibited significant variability (Fig. 1). The maximum damage recorded was ₹57,291.10 crore in 2015, while the minimum was ₹7.14 crore in 1965. Over this 68-year period, the average annual damage was approximately ₹6,428.67 crore. The highest flood damage in India can be attributed to several factors, such as extreme rainfall events and severe flooding, which are the primary causes of such extensive damage. India is highly vulnerable to floods, with approximately 80% of the total annual rainfall concentrated during the monsoon season (June–September), resulting in very high river discharges and extensive damage to life and property^84^. The frequency of extreme floods has been increasing in India, with both flood events and fatalities increasing over time^23^. Interestingly, although severe flood events account for only 19% of total flood occurrences, they are responsible for 56% of flood fatalities^23^. This suggests that the most damaging floods are often caused by extreme weather events. The 2015 floods likely fall into this category of severe events, explaining the exceptionally high damage recorded in that year. The high damage can also be attributed to the increased exposure of assets and populations in flood-prone areas. Approximately 28.13% of India’s built-up area falls within highly susceptible flood zones, including major cities^85^. Additionally, a significant portion of the country’s agricultural land is at risk, particularly in the Brahmaputra, Ganga, and Indus River basins^85^. This combination of urban and agricultural vulnerabilities contributes to the potential for massive economic losses during severe flood events. This wide range highlights the increasing vulnerability and exposure of infrastructure and agriculture to flood damage and possibly improved reporting mechanisms. The trend reveals a clear escalation in flood damages, especially from the late 1990s. Notable spikes occurred in 2009, 2013, and 2015, each surpassing ₹30,000 crore, indicating years of severe natural disasters and extreme weather events. In contrast, the earlier decades, particularly the 1950s and the 1960s, saw relatively modest figures, often below ₹100 crore. This could reflect both fewer extreme events and limited infrastructure at risk in these areas. The sharp rise in recent decades underscores the growing economic impact of climate-related events and the need for enhanced disaster preparedness and mitigation strategies to reduce economic losses.

#### Actual/observed rainfall

**Fig. 2:**
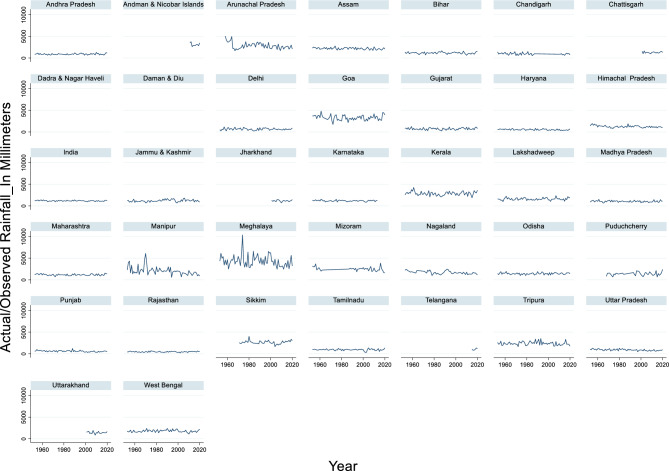
Actual/observed rainfall_in millimeters.

The time series data of rainfall in India from 1953 to 2020 revealed notable fluctuations in annual precipitation levels (Fig. 2). The maximum rainfall recorded during this period was 1401.4 mm in 1990, while the minimum rainfall was 930.1 mm in 2002. The average annual rainfall across these 68 years was approximately 1172.59 mm. These figures highlight the variability in India’s monsoon patterns, which are critical for the country’s agriculture and water resources. This variability underscores the importance of long-term climate monitoring and adaptive planning in the region. Years with high rainfall, such as 1990, may lead to flooding and waterlogging, whereas low-rainfall years, such as 2002, can result in droughts and water scarcity. These extremes can significantly impact crop yields, water supply, and overall economic stability.

The relationship between actual/observed rainfall and total flood damage is complex and multifaceted, as evidenced by the research papers provided. High rainfall amounts are strongly correlated with increased flood damage to crops, houses, and public utilities. Tarhule^86^ reported that in Niger, 79 damaging rainfall and flood events destroyed 5,580 houses and rendered 27,289 people homeless between 1970 and 2000. Similarly, Chowdhury and Hassan^87^ described how unusually high rainfall (250–450% above normal) in Bangladesh during the late monsoon of 2000 led to extensive flooding and crop damage. However, the relationship between rainfall and flood damage is not linear. Davenport et al. in their study found that, the precipitation changes contributed to approximately one-third of the cumulative flood damages in the United States from 1988 to 2017, totalling $73 billion^88^. This suggests that although rainfall is a significant factor, other variables also play important roles in determining flood impacts. Interestingly, the timing and intensity of rainfall, rather than the total amount, can significantly influence flood damage. Wei and the co-authors emphasized the importance of predicting rainfall patterns in the first 20 min to assess flood risk^89^. Additionally, Wu et al. during 2012 noted that in Shanghai, while annual rainfall totals showed no significant trends, increases in seasonal and monthly rainfall intensities contributed to a greater waterlogging risk^90^.

#### Mangrove cover

**Fig. 3:**
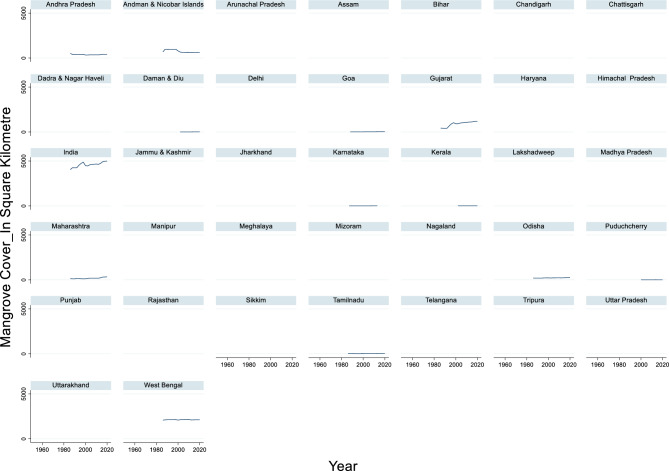
Mangrove Cover_in Square Kilometre.

The time-series data of mangrove cover in India from 1986 to 2020 revealed significant changes in the extent of mangrove forests (Fig. 3). The maximum mangrove cover recorded during this period was 4992 square kilometers in the year 2020, while the minimum mangrove cover was 4046 square kilometers in 1986. The average mangrove cover across these years was approximately 4588.83 square kilometers. These figures indicate a general trend of increasing mangrove cover over the years, which is a positive sign for coastal ecosystem health and biodiversity in the region. Mangroves play a crucial role in protecting coastlines from erosion, providing habitats for diverse marine life, and sequestering carbon. The observed increase in mangrove cover suggests successful conservation and restoration efforts have been undertaken.

Mangrove cover significantly reduces total flood damage to crops, houses, and public utilities, as evidenced by several studies. Mangrove forests provide crucial ecosystem services, particularly in coastal areas, by acting as natural barriers against cyclones and floods^91^. In a study conducted in the Bhitarkanika mangrove ecosystem in India, villages protected by mangroves experienced significantly lower damage costs than unprotected villages. The loss incurred per household in the mangrove-protected village was US$ 33.31, while it was US$ 153.74 in the village with an embankment but no mangrove protection^91^. The protective function of mangroves is further supported by global-scale research on tropical cyclones and mangrove forests. Stands with higher aboveground biomass experience decreased canopy damage during cyclone events^92^. This suggests that denser mangrove cover provides better protection against flood-related damage. However, the effectiveness of mangrove cover for flood protection can vary based on forest structure and size. The spatial heterogeneity of forest structures affects the capacity for wave or surge attenuation, which directly impacts flood damage reduction^93^. Additionally, fragmented or restored mangroves may provide less protection than old, contiguous mangrove patches, as evidenced by the lower biomass carbon density in fragmented areas^94^.

#### Per capita NSDP

**Fig. 4:**
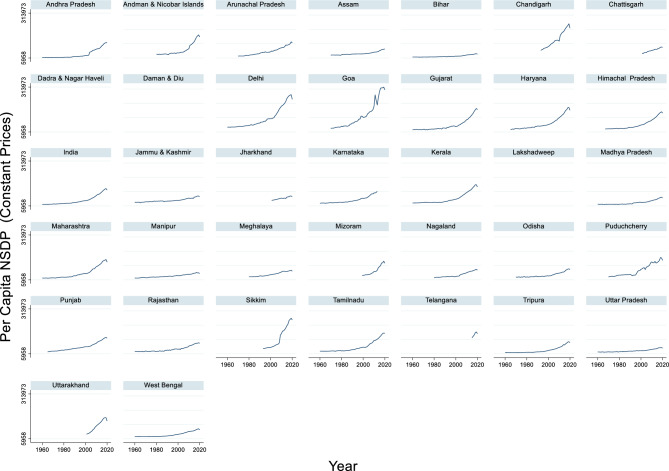
Per Capita NSDP (Constant Prices).

The time series data of India’s per capita Net State Domestic Product (NSDP) at constant prices from 1960 to 2020 reveal significant economic growth over the decades (Fig. 4). The maximum per-capita NSDP recorded during this period was ₹124,779 in 2019, while the minimum per-capita NSDP was ₹15,892 in 1965. The average per-capita NSDP across these years was approximately ₹42,760.79. These figures indicate a general trend of increasing economic prosperity, reflecting improvements in productivity, infrastructure, and overall standard of living. Zhai et al.^95^ noted that household income significantly affects both house and content damage values in flood events. This suggests that economic factors at the individual level can impact the extent of flood damage. This upward trend in per capita NSDP underscores the positive impact of economic reforms, industrialization, and technological advancements in India. The substantial growth observed in recent years, particularly from 2000 onwards, highlights the country’s progress in various sectors, such as information technology, manufacturing, and services.

#### Forest area

**Fig. 5:**
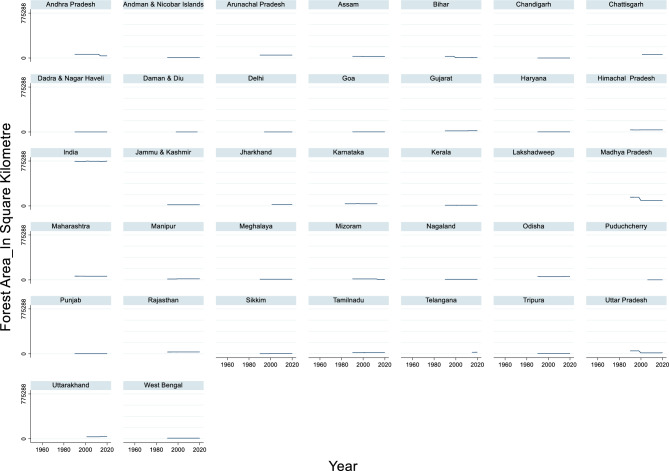
Forest Area_in Square Kilometre.

The time-series data of India’s forest area from 1990 to 2020 revealed notable fluctuations in the extent of forest cover (Fig. 5). The maximum forest area recorded during this period was 775,288 square kilometers in the year 2020, while the minimum forest area was 764,566 square kilometers in 2014. The average forested area across these years was approximately 768,910.33 square kilometers. These figures indicate a general trend of maintaining substantial forest cover over the years, which is crucial for biodiversity, climate regulation, and ecological balance.

Despite these fluctuations, the overall trend suggests efforts towards forest conservation and reforestation. The increase in forest area observed in 2020 highlights the successful initiatives and policies aimed at preserving and expanding forested regions. However, continuous monitoring and adaptive management are essential to address challenges such as deforestation, land-use change, and climate impact.

Forest areas have a significant impact on total flood damage, affecting crops, houses, and public utilities in several ways. Forest cover was negatively correlated with flood frequency and severity. A study across 56 developing countries found that flood frequency decreased with greater natural forest area and increased with forest loss^96^. This relationship was maintained even after controlling for factors such as rainfall, slope, and degraded landscape area. The model predicted that a 10% decrease in natural forest area could increase flood frequency by 4–28% among the studied countries. Forests play a crucial role in flood protection by enhancing water retention and infiltration. A study in Flanders, Belgium, demonstrated that afforestation of 187.5 ha (3.9% of the catchment area) resulted in a 57% reduction in flood risk, equivalent to €100,000 in avoided damage^97^. This highlights the potential of nature-based solutions, such as strategic forest placement, for mitigating flood risk. Interestingly, the impact of forest cover on flood damage varies depending on the scale and context. While global patterns show a clear correlation between forest loss and increased flood risk, this relationship may be more complex at the local level. For instance, some studies have focused on other factors, such as flood depth, building characteristics, and socioeconomic variables, when assessing flood damage^98–100^.

#### Total population

**Fig. 6:**
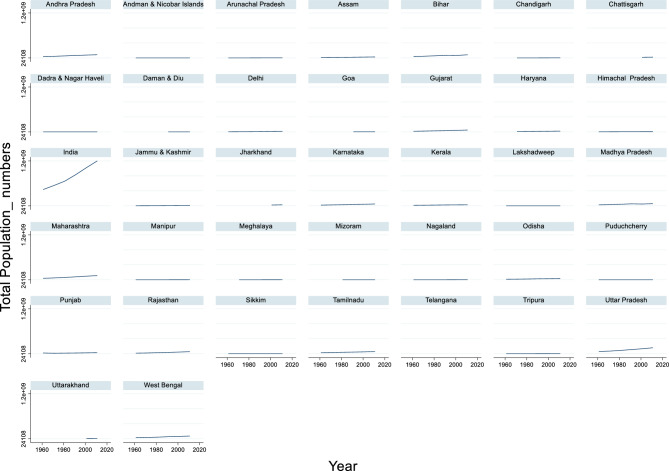
Total Population_ in numbers.

The time series data of India’s total population from 1961 to 2011 revealed significant growth over the decades (Fig. 6). The maximum population recorded during this period was 1,210,854,977 in 2011, while the minimum population was 439,235,082 in 1961. The average population across these years stands at approximately 788,420,339. These figures reflect a consistent upward trend in the population, driven by improvements in healthcare, reduced mortality rates, and enhanced living standards.

This steady population growth presents both opportunities and challenges for the country. A growing population can contribute to a larger workforce, increased domestic demand, and greater cultural vibrancy. However, it also intensifies the pressure on natural resources, infrastructure, and public services. To harness the benefits of this demographic expansion while mitigating its challenges, India must continue to invest in education, healthcare, employment generation, and sustainable urban planning.

Population density and urbanization may indirectly influence flood damage. Collins et al.^101^ mentioned that high urban road density is associated with higher flood damage probabilities. This suggests that areas with higher population concentrations may experience greater flood damage owing to increased infrastructure and exposure. Interestingly, Ten Veldhuis^102^ presented a different perspective on damage assessment. It compares monetary values with the number of people affected by floods. The study found that when damage is expressed in terms of the number of people affected, road flooding becomes the main contributor to total flood damage, as opposed to building damage when assessed monetarily.

#### Area affected due to flood

**Fig. 7:**
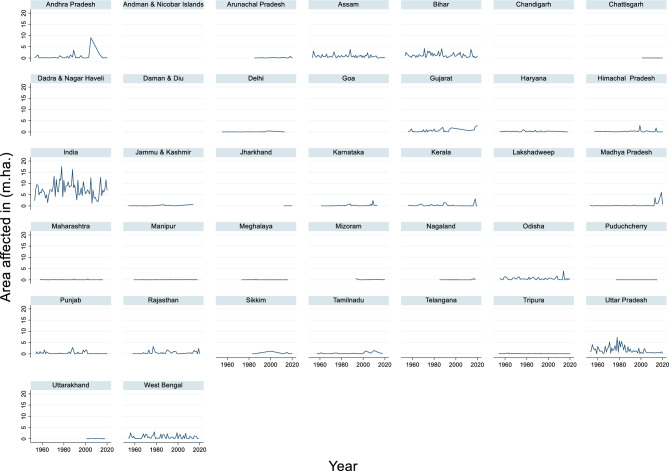
Area affected due to flood in (m.ha.)

The time series data of the area affected in India from 1953 to 2020 revealed significant fluctuations in the extent of the affected regions (Fig. 7). The maximum area affected during this period was 17.5 million hectares in 1978, while the minimum area affected was 1.096 million hectares in 2006. The average area affected across these years was approximately 7.244 million hectares. These figures highlight the variability in the impact of adverse events, such as droughts, floods, or pest outbreaks, on land over decades.

This variability underscores the importance of long-term monitoring and adaptive planning. Years with highly affected areas, such as 1978, can severely disrupt agriculture, food security, and rural livelihoods, while years with lower impact, such as 2006, may reflect effective disaster management or favorable environmental conditions.

The area affected by floods has a significant impact on the total flood damage to crops, houses, and public utilities. As the inundated area increases, the extent of damage typically increases proportionally. In the Kopili River Basin, approximately 3.89 lakh hectares (29% of the basin area) were affected by floods in 1977, 1988, and 1998–2015^103^. This extensive flooding led to the categorization of more than 150 villages in high-to very-high flood hazard zones, indicating substantial damage to crops, houses, and infrastructure in these areas. The 2010 floods in Pakistan’s Central Indus Basin demonstrated how a large affected area can result in severe economic losses for the country. The total estimated economic loss was approximately 2.54 million US$, with infrastructure being the most affected sector (1.65 million US$), followed by agriculture^104^. Similarly, the 2021 floods in the Netherlands caused damage estimated at € 400–500 million, affecting more than 2,500 houses, 5,000 inhabitants, and 600 businesses^105^. The maximum area affected by floods in India during this period was 17.5 million hectares in 1978. Although the provided context does not explicitly state the reason for this peak, it can be inferred that 1978 likely experienced exceptionally heavy monsoon rains or extreme weather events. The Brahmaputra River, which floods annually, significantly contributes to India’s flood-prone areas. In 2003, satellite data analysis revealed that 25–30% of the area in some districts was flood-affected during July and August^106^, highlighting the recurring nature of large-scale flooding in certain regions. In conclusion, the area affected by floods was directly correlated with total flood damage.

#### Damage to crops area due to flood

**Fig. 8:**
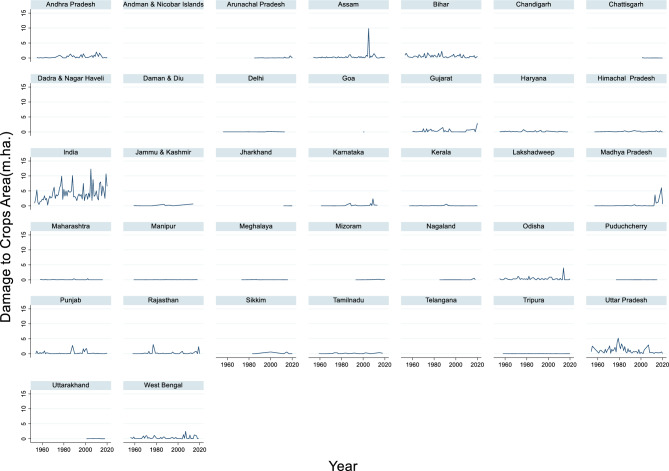
Damage to Crops Area due to flood (m.ha.)

The time series data of the crop-damaged area in India from 1953 to 2020 revealed significant fluctuations in the extent of the affected regions (Fig. 8). The maximum crop damage area during this period was 12.299 million hectares in 2005, while the minimum crop damage area was 0.27 million hectares in 1965. The average crop damage area across these years was approximately 4.055 million hectares. These figures highlight the variability in the impact of adverse events, such as droughts, floods, or pest outbreaks, on agricultural land over decades.

This variability underscores the importance of long-term monitoring and adaptive planning. Years with high crop damage areas, such as 2005, can severely disrupt agriculture, food security, and rural livelihoods, whereas years with lower impact, such as 1965, may reflect effective disaster management or favorable environmental conditions.

Several studies have discussed crop damage due to flooding, but they have focused on different regions and time periods. For example, Mohammadi et al.^107^ estimates potential production losses of rice and wheat in flood risk zones in northern Iran. Rahman et al.^108^ presented a method for assessing crop-specific damage immediately after flood events using a Disaster Vegetation Damage Index (DVDI) for cases in Iowa, Nebraska, and Texas. These papers highlight the various factors contributing to crop damage during floods. These include flood depth, flow velocity, season of flood occurrence, and proximity to water bodies^101,109^. Additionally, the duration of flooding plays a crucial role in determining the extent of crop damage, especially in areas with multi-cropping practices, such as the middle and lower reaches of the Yangtze River Basin^110^. Although the specific reason for the maximum crop damage area of 12.299 million hectares in 2005 is not provided, factors such as extreme rainfall events, river overflow, and inadequate flood management infrastructure could contribute to such extensive damage.

#### Cattle lost due to flood

**Fig. 9:**
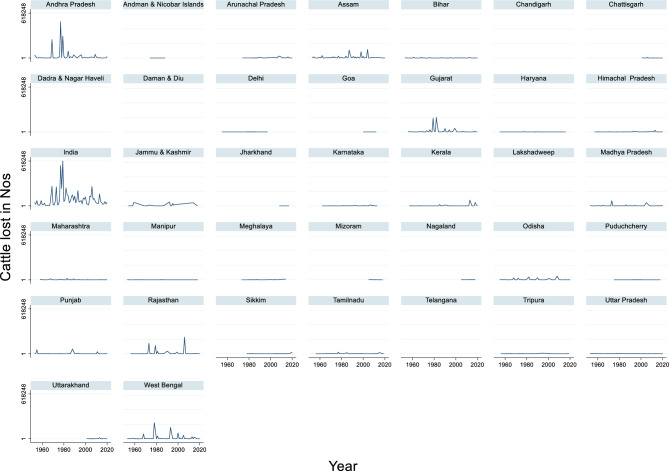
Cattle lost due to flood in Nos.

The time series data of cattle loss in India from 1953 to 2020 revealed significant fluctuations in the number of cattle lost due to various adverse events (Fig. 9). The maximum cattle loss during this period was 618,248 in 1979, while the minimum cattle loss was 4,572 in 1963. The average cattle loss across these years was approximately 104,987. These figures highlight the variability in the impact of adverse events, such as natural disasters, diseases, or other calamities, on livestock over decades.

This variability underscores the importance of long-term monitoring and adaptive planning. Years with high cattle losses, such as 1979, can severely disrupt rural livelihoods and agricultural productivity, whereas years with a lower impact, such as 1963, may reflect effective disaster management or favorable environmental conditions.

Floods can cause significant damage to agricultural areas, including potential impacts on livestock health. Kok et al.^105^ mentioned that the 2021 floods in the Netherlands affected crops, though it does not specifically mention cattle losses. Similarly, Samuele et al.^111^ discusses flood impacts on agricultural areas in Italy, focusing on crop damage rather than livestock. The economic impact of floods can be substantial and varied. Mahmood et al.^104^ reported that the 2010 flood in Pakistan caused an estimated economic loss of approximately 2.54 million US$, with agriculture being the second most affected sector after infrastructure. This suggests that floods can have a significant impact on rural areas where cattle farming is prevalent. It is important to note that the impacts of flooding can vary greatly depending on factors such as flood severity, local topography, and preparedness measures. Collins et al.^101^ mentioned that flood damage probability is influenced by factors such as elevation, proximity to streams, extreme precipitation, and urban road density.

#### Human lives lost due to flood

**Fig. 10:**
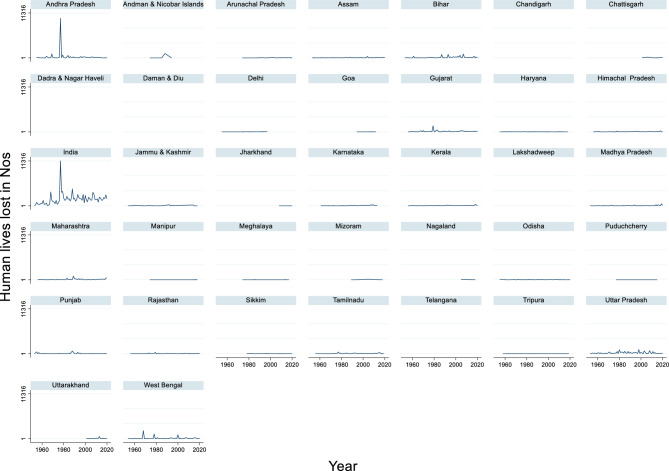
Human lives lost due to flood in Nos.

The time-series data of human lives lost in India from 1953 to 2020 revealed significant fluctuations in the number of lives lost owing to various adverse events (Fig. 10). The maximum human lives lost during this period was 11,316 in 1977, while the minimum human lives lost was 37 in 1953. The average human lives lost across these years is approximately 1,675.63. These figures highlight the variability in the impact of adverse events, such as natural disasters, diseases, or other calamities, on human lives over decades.

This variability underscores the importance of long-term monitoring and adaptive planning. Years with high human lives lost, such as 1977, can severely disrupt communities and overall societal stability, while years with lower impact, such as 1953, may reflect effective disaster management or favorable environmental conditions.

According to Singh and Kumar^23^, between 1978 and 2006, flood events in India claimed approximately 44,991 lives, with an average of 1,551 lives lost each year. This translates to a loss of 1.5 human lives per million population. The majority (56%) of flood fatalities were caused during severe flood events, although these events accounted for only 19% of the total flood occurrences. Regarding total flood damages, India suffered a cumulative flood-related economic loss of approximately 16 billion US$ between 1978 and 2006, with a maximum economic loss of 1.6 billion US$ in the year 2000 alone^23^. These damages include impacts on crops, houses, and public utilities. The authors also suggested that both flood events and fatalities have increased in India over time, which could explain the high number of casualties in 1977. In conclusion, flood-related fatalities and economic damage in India are significant and widespread.

#### Capital expenditure

**Fig. 11:**
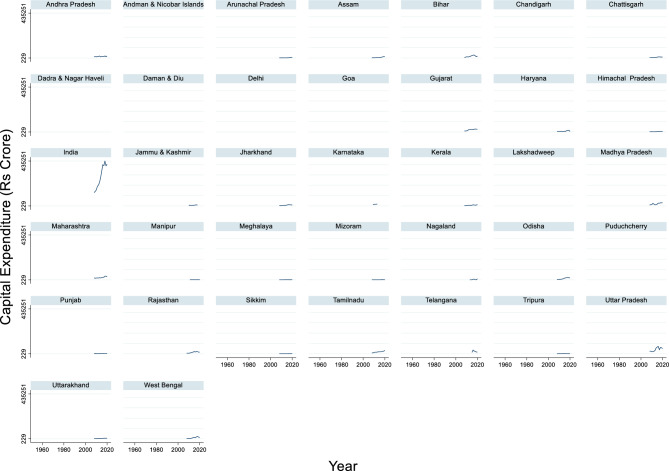
Capital expenditure (Rs Crore).

The time series data of India’s capital expenditure from 2008 to 2020 show a consistent upward trend, reflecting the government’s increasing investment in infrastructure and development projects (Fig. 11). The maximum capital expenditure during this period was ₹4,35,251 crore in 2018, while the minimum was ₹1,28,957 crore in 2008. The average capital expenditure across these years was approximately ₹2,82,234 crore. This steady rise indicates a strategic focus on long-term economic growth through capital formation and the creation of public assets.

This growth in capital expenditure aligns with India’s broader economic goals, including the modernization of transport, energy, and digital infrastructure. The sharp increase post-2014 suggests a policy shift toward boosting public investment to stimulate economic activity, especially in the face of global economic uncertainties. Sustained capital spending is crucial for job creation, enhancing productivity, and improving quality of life.

Capital expenditure on flood protection measures tends to increase in response to greater flood damages. The authors Ishiwatari & Sasaki^112^ found that "greater flood damage is associated with a larger budget for flood protection". Governments typically increase budgets for flood protection after major disasters occur and further increase them as GDP per capita and population density rise. This suggests a reactive approach to flood damage mitigation through capital expenditures. Interestingly,^113^ found a negative relationship between capital expenditure on human capital development (including education and health) and economic output levels in Nigeria. Although not directly related to flood damage, this highlights that capital expenditure does not always translate to positive economic outcomes. This finding contradicts the general assumption that increased capital spending leads to better outcomes.

#### Revenue expenditure

**Fig. 12:**
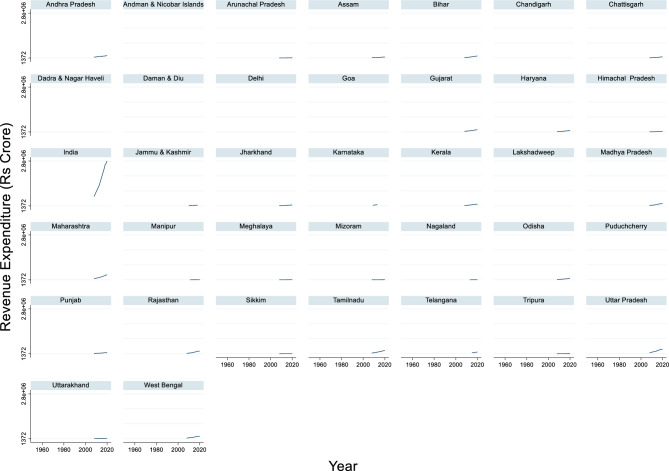
Revenue Expenditure (Rs Crore).

The time series data of India’s revenue expenditure from 2008 to 2020 show a consistent upward trend, reflecting the government’s increasing spending on public services, subsidies, and administrative costs (Fig. 12). The maximum revenue expenditure during this period was ₹28,03,731 crore in 2020, whereas the minimum was ₹5,87,373 crore in 2008. The average revenue expenditure across these years was approximately ₹16,49,344 crore. This steady rise indicates a growing commitment to welfare programs, infrastructure development, and economic stimulus.

This growth in revenue expenditure aligns with India’s broader economic goals, including poverty alleviation, healthcare, and education. The sharp increase post-2014 suggests a policy shift toward boosting public investment to stimulate economic activity, especially in the face of global economic uncertainties.

Governments tend to increase their budgets for flood protection in response to greater flood damage^112^. This suggests a positive correlation between flood damage and subsequent revenue expenditures for flood protection measures. The study found that governments typically start increasing budgets after major disasters and further increase them as GDP per capita and population density rise. This reactive approach to flood protection investment highlights the need for more proactive strategies for disaster risk reduction. Interestingly, there is evidence of a causal relationship between government expenditure and revenue. The authors Hondroyiannis and Papapetrou^114^ suggest that for Greece, over the period 1957–1993, there was a long-run relationship between government spending and revenue, with expenditures causing revenues. This implies that increased spending on flood protection could potentially lead to increased government revenue in the long term, possibly through economic stability and growth resulting from improved disaster preparedness. The relationship between revenue expenditure and flood damage is not straightforward, as it involves various factors, such as the level of flood protection, socioeconomic development, and climate change scenarios^115,116^. For instance,^116^ demonstrated that autonomous adaptation can significantly reduce potential flood consequences, mitigating approximately half of the potential increase in economic losses by 2100. This suggests that strategic revenue expenditure on adaptation measures can substantially reduce future flood damage.

#### Government expenditure on account of natural calamities

**Fig. 13:**
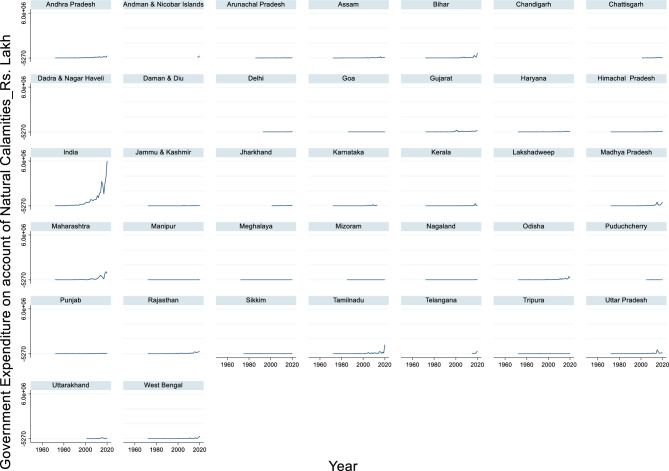
Government Expenditure on account of natural calamities (Rs.) Lakh.

The time series data of India’s government expenditure on natural calamities from 1972 to 2020 shows a significant upward trend, reflecting the increasing financial burden of natural disasters over the decades (Fig. 13). The maximum expenditure during this period was ₹60,13,579 lakhs in 2020, while the minimum expenditure was ₹8,475 lakhs in 1976. The average expenditure across these years was approximately ₹7,34,431 lakhs. This dramatic rise underscores the escalating costs associated with disaster response and recovery, which are driven by both the frequency and severity of natural calamities.

This trend highlights the growing vulnerability of populations and infrastructure to climate-related events. The sharp increase in expenditure, particularly in the last two decades, suggests a shift in policy focus toward more comprehensive disaster relief and rehabilitation efforts. While this reflects a proactive approach to disaster management, it also emphasizes the need for long-term investments in climate resilience, sustainable development, and risk reduction strategies.

Government expenditure on natural calamities, particularly floods, is often reactive and increases in response to disasters. Miao et al.^117^ find that natural disasters, including floods, increase a province’s total governmental spending and intergovernmental revenues received from the central government. Similarly,^112^ report that "greater flood damage is associated with a larger budget for flood protection" and that "governments start increasing budgets after major disasters happen". Interestingly, the impact of government expenditure on flood damage mitigation has shown mixed results. Miao et al.^117^ suggests that fiscal decentralization in natural resource expenditures, which includes flood control measures, may lead to inefficient protection against natural disasters, resulting in greater economic losses from floods and storms. This finding contradicts the expected outcome of increased government spending on disaster mitigation.

#### Population affected due to flood

**Fig. 14:**
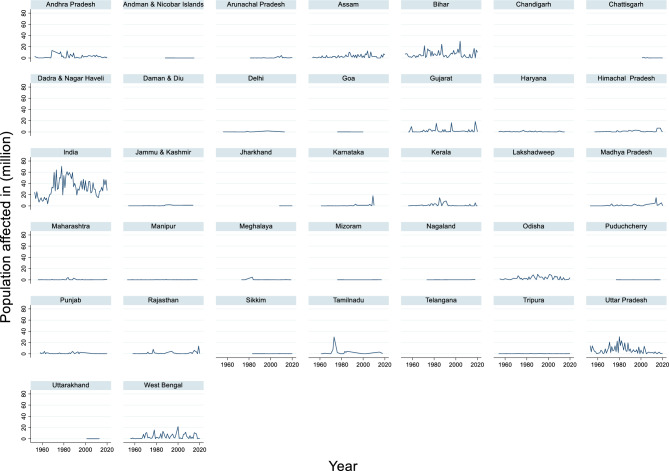
Population affected due to flood in (million).

The time-series data of the population affected by flood damage in India from 1953 to 2020 revealed significant fluctuations in the number of affected individuals (Fig. 14). The maximum population affected during this period was 70.45 million in 1978, while the minimum population affected was 3.61 million in 1965. The average population affected across these years was approximately 32.34 million. These figures highlight the wide-ranging impact of natural disasters, such as floods, droughts, and cyclones, on the Indian population over decades.

This variability emphasizes the need for robust disaster preparedness and response systems in the region. Years with high numbers of affected individuals, such as 1978, often correspond to large-scale disasters that strain resources and infrastructure, whereas years with lower figures may reflect either fewer disasters or more effective mitigation strategies.

#### Damage to houses due to flood

**Fig. 15:**
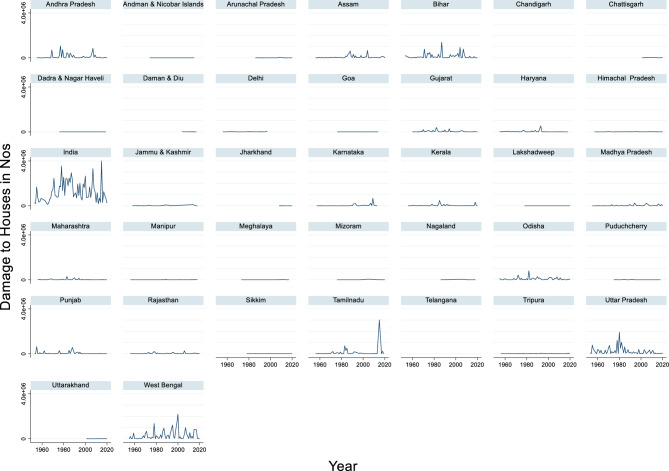
Damage to Houses due to flood in Nos.

The time-series data of damage to houses in India from 1953 to 2020 revealed substantial variation in the number of houses affected by flood damage (Fig. 15). The maximum damage occurred in 2015, with 3,959,191 houses damaged, whereas the minimum damage was recorded in 1965, with 112,957 houses affected. The average number of houses damaged annually over this period stands at approximately 1,213,606. These figures reflect the varying intensity and frequency of natural disasters, such as floods, cyclones, and earthquakes, across different years.

The sharp spikes in certain years, such as 1978, 2000, 2007, and 2015, suggest the occurrence of particularly severe events that caused widespread destruction. Conversely, years with lower figures may indicate fewer disasters or more effective disaster mitigation and preparedness.

Floods in India have a substantial impact on the population, causing widespread displacement, loss of life, and economic damage to the population. In the Ganga–Brahmaputra Basin, one of the most flood-prone regions in India, a recent study estimated that 23.29 million people were affected by floods^118^. The impact is particularly severe in densely populated areas such as Bihar and Bangladesh, where people often reside near riverbanks because of their dependence on river water^118^. Urban areas are also increasingly vulnerable, with more than half of India’s population expected to migrate to urban regions by 2050, thereby exacerbating the risk of urban flooding^119^. The relationship between the population affected and flood damage is not always linear. Factors such as socioeconomic status, housing quality, and access to resources play a crucial role in determining vulnerability. For instance, rural households in India are highly vulnerable to flood hazards because of their dependence on natural resources for livelihood and poor socioeconomic situations^49^. Additionally, certain demographic characteristics, such as low literacy rates, high dependency ratios, and weak housing structures, increase residents’ vulnerability to flood damage^49^. In conclusion, the population affected by floods in India is closely linked to the extent of the flood damage. However, this relationship is influenced by various factors, including urbanization, socioeconomic conditions, and geographical location. Understanding these complex interactions is crucial for developing effective flood risk reduction strategies and management policies in India.

#### Damage to public utilities due to flood

**Fig. 16:**
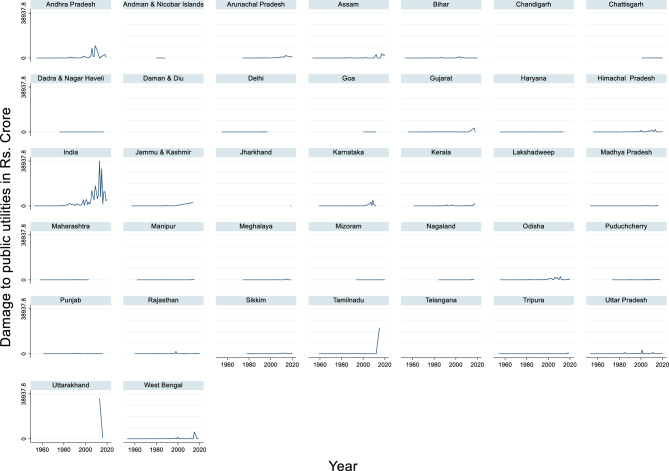
Damage to public utilities due to floods in Rs. Crore.

The time series data on damage to public utilities in India from 1953 to 2020 reveal a dramatic escalation in financial losses over the decades (Fig. 16). The maximum damage was recorded in 2013, amounting to ₹38,937.84 crore, while the minimum was ₹1.05 crore in 1956. The average damage across these years was approximately ₹3,443.37 crore. This sharp rise in damage reflects the increasing scale and intensity of natural disasters and the growing complexity and value of public infrastructure exposed to such events.

The data underscore a critical shift in the nature of disaster impacts, from localized, lower-cost damage in the mid-twentieth century to widespread, high-cost disruptions in recent decades. This trend is likely driven by rapid urbanization, climate change, and infrastructure network expansion. The surge in expenditures, especially post-2000, signals a growing recognition of the need for robust disaster-response mechanisms.

Economic development plays a crucial role in mitigating flood damage, including damage to public utilities and infrastructure. As states develop economically, they are better equipped to invest in flood prevention measures and improve their infrastructure resilience^25^. However, the increasing trend of urban flooding in India poses a major threat to public utilities in cities where population density is high and infrastructure is concentrated^119^. Interestingly, although structural measures such as embankments and dams have been implemented to protect public utilities and other assets, statistics do not reveal a major reduction in flood damage^84^. This suggests that a more comprehensive approach, including both structural and nonstructural measures, may be necessary to effectively protect public utilities and reduce overall flood damage. In conclusion, damage to public utilities is a significant component of the overall flood damage in India. Effective flood management requires a combination of economic development, political coordination^25^, and integrated approaches that consider both structural and nonstructural measures^84^. Additionally, the use of social and remote sensing technologies^120,121^ could help in the rapid assessment and response to flood-related damage to public utilities, potentially reducing the overall impact of floods on infrastructure and services.

### Determinants of total flood damage in India

#### Panel data description

The dataset exhibits a robust panel structure, encompassing data from 36 Indian states and union territories or regions over a substantial time span of 1953 to 2020 (Table 2). This resulted in a total of 2,235 observations, with an average of approximately 62 time periods per state. Such long and rich temporal coverage makes the dataset particularly well-suited for panel regression models, enabling the analysis of both cross-sectional and longitudinal dynamics.

**Table 2:** Summary statistics for panel data.

Variable		Mean	Std. dev	Min	Max		Observations
State	Overall	18.077	10.207	1	36		N = 2235
	Between		10.536	1	36		n = 36
	Within			18.077	18.077	T	bar = 62.083
Year	Overall	1987.132	19.771	1953	2020		N = 2235
	Between		8.326	1983	2017.5		n = 36
	Within		19.310	1953.632	2020.632	T	bar = 62.083
$TD$	Overall	377.179	1992.714	0.001	39,541		N = 1109
	Between		3383.540	0.099	20,164.62		n = 35
	Within		1772.000	− 18,999.200	25,383.020	T	bar = 31.686
$CE$	Overall	10,195.430	11,025.880	229	72,275		N = 337
	Between		9533.508	896.8462	42,310.08		n = 28
	Within		5635.018	− 12,159.650	40,160.350	T	bar = 12.036
$RE$	Overall	60,592.820	60,971.230	1372	308,564		N = 337
	Between		50,961.440	3845.538	187,374.1		n = 28
	Within		33,174.630	− 51,190.250	187,025.000	T	bar = 12.036
$GENC$	Overall	27,328.900	81,989.510	− 5270	1,202,170		N = 1287
	Between		41,269.910	354.3143	179,525.2		n = 32
	Within		76,403.330	− 101,788.600	1,165,346.000	T	bar = 40.219
$DTH$	Overall	72,449.020	203,269.600	1	3,015,270		N = 1141
	Between		69,376.190	69	304,023.3		n = 34
	Within		186,224.700	− 231,349.300	2,948,157.000	T	bar = 33.559
$DTPU$	Overall	253.880	1566.119	0.001	34,977		N = 912
	Between		3005.179	0.006	17,875.82		n = 35
	Within		1307.307	− 16,847.300	22,458.190	T	bar = 26.057
$PA$	Overall	4.356	9.436	0.001	70.45		N = 1009
	Between		5.676	0.01	32.3351		n = 33
	Within		5.370	− 24.369	42.471	T	bar = 30.576
$ARF$	Overall	1587.084	1019.632	175.6	10,367.4		N = 1907
	Between		914.028	461.6956	4318.91		n = 34
	Within		425.385	− 283.427	7635.574	T	bar = 56.088
$TP$	Overall	25,700,000	33,300,000	24,108	2.00E + 08		N = 184
	Between		29,800,000	45,499.5	1.30E + 08		n = 35
	Within		12,600,000	− 30,200,000	95,800,000		T = 5.25714
$PCNSDP$	Overall	47,699.670	44,103.760	5958	313,973		N = 1596
	Between		31,470.550	12,309.49	134,348		n = 33
	Within		35,536.950	− 40,082.290	249,418.700	T	bar = 48.364
$MC$	Overall	443.240	633.146	1	2155		N = 176
	Between		624.368	1.4	2116.294		n = 12
	Within		104.483	− 36.231	743.769	T	bar = 14.667
$FA$	Overall	23,585.770	25,163.940	0.7	155,414		N = 489
	Between		24,413.610	5.881818	110,769.9		n = 35
	Within		6109.540	4116.306	68,229.910	T	bar = 13.971
$AA$	Overall	0.530	0.911	0.001	9.04		N = 908
	Between		0.407	0.0146	1.764733		n = 30
	Within		0.776	− 1.181	8.993	T	bar = 30.267
$DTCA$	Overall	0.310	0.655	0.001	9.84		N = 895
	Between		0.236	0.0035	1.114228		n = 31
	Within		0.596	− 0.763	9.742	T	bar = 28.871
$CL$	Overall	6256.391	27,550.530	1	500,978		N = 988
	Between		6745.107	2.333333	33,024.54		n = 32
	Within		26,504.570	− 26,764.150	474,209.800	T	bar = 30.875
$HLL$	Overall	108.312	364.549	1	9974		N = 1044
	Between		93.705	3.166667	368.25		n = 33
	Within		353.400	− 258.938	9756.985	T	bar = 31.636

The dependent variable, representing the total damage to crops, houses, and public utilities (in ₹ crore), has a mean value of ₹377.18 crore and a maximum value of ₹39,541 crore. The within-state variation is considerable, ranging from -₹18,999 crore to ₹25,383 crore, indicating significant year-to-year fluctuations in disaster-related damage. The between-state variation is also notable, suggesting that certain states are more consistently affected by high levels of damage, likely because of geographic and socioeconomic vulnerabilities.

Among the fiscal variables, the average capital expenditure is ₹10,195 crore, with values ranging from ₹229 crore to ₹72,275 crore. The within variation (T̄ = 12.04) reflects substantial changes in capital investment over time, within states. Revenue expenditure is even higher, averaging ₹60,593 crore and peaking at ₹3,08,564 crore, again showing strong within-variation. Government expenditure on flood damage, reported in ₹ lakh, averages ₹27,329 lakh and reaches over ₹12,000 crore. This variable exhibits high within-variation (T̄ = 40.2), indicating its reactivity to disaster events.

Damage indicators further illustrate the physical impacts of disasters. Damage to houses averages 72,449 units, with a maximum of over 3 million, and shows significant within-variation, highlighting the episodic but severe nature of housing losses. Damage to public utilities averages ₹253.88 crore, with a maximum of ₹34,977 crore, also showing high within variation. These figures underscore the variability and intensity of disaster impact across time and space.

Demographic and socioeconomic variables provide additional information. The population affected by disasters averages 4.36 million, with a maximum of 70.45 million people. The wide within variation (T̄ = 30.6) reflects the varying scale of disasters and their toll on humans. The total population averages 25.7 million and reaches up to 200 million, with high within-variation capturing demographic growth and migration. Per capita NSDP (at constant prices) averages ₹47,700 and peaks at ₹3,13,973, with large within-state variation (T̄ = 48.4), indicating economic development trends within states.

Environmental variables, such as mangrove and forest cover, exhibit more structural characteristics. Mangrove cover averages 443 sq. km, with a maximum of 2,155 sq. km, and is dominated by between-state variation, suggesting that it is largely a fixed geographic feature. The forest area averages 23,586 sq. km and reaches up to 1,55,414 sq. km, with both between and within variation, indicating some changes over time but primarily reflecting geographic differences.

Climate and agricultural impact variables also play crucial roles. The actual rainfall averages 1,587 mm, with a maximum of 10,367 mm, and shows substantial within variation, reflecting annual monsoon fluctuations. Damage to crops averages 0.31 million hectares, with a maximum of 9.84 million hectares, and the area affected shows similar patterns. These variables are essential for understanding the agricultural impacts of disasters.

Finally, livestock and human losses provide insights into the broader social impacts. Cattle lost averages 6,256 animals, with a maximum of 500,978, and showed high within variation, indicating vulnerability to disaster events. Human lives lost averaged 108, with a maximum of 9,974, and also exhibited significant within-variation, underscoring the human cost of disasters across states and years.

In summary, the dataset is comprehensive and well-structured for panel data analysis, with a strong mix of between- and within-variation across fiscal, demographic, environmental, and impact-related variables. Its extensive temporal and geographic coverage supports nuanced empirical analyses of disaster vulnerability, resilience, and the effectiveness of policy responses across Indian states.

#### Workflow of the study

This study aimed to assess the socioeconomic and environmental determinants of flood vulnerability in India using a panel data approach. After collecting the data, we performed cleaning and interpolation to address missing values, as missing values affect the results^122^. We then conducted panel unit root tests for stationarity and checked for the presence of multicollinearity. Panel unit root tests for stationarity and multicollinearity are crucial in econometric analysis because they help determine the appropriate modeling approach^123^. To determine the appropriate model, we tested for endogeneity. If endogeneity exists, we must use IV/2SLS/GMM models; otherwise, we use FE/RE models^124^. Since we do not have endogeneity, we proceeded with the Fixed Effects (FE) and Random Effects (RE) models. A Hausman test was used to select between them^125^. Finally, we conducted diagnostic and robustness checks, including the Wooldridge test for serial correlation and cluster-robust standard errors for the RE model.

#### Stationarity check (panel unit root tests)

In the Fisher-type panel unit root test, several statistics were used to assess stationarity. The P (inverse chi-squared) is best when the number of panels (N) is small, whereas the Z (inverse normal) is suitable for large N. The L* (inverse logit t) is robust when both N and T (time periods) are moderate, and the Pm (modified inverse chi-squared) is preferred when T is large. In our case, with an average of 36 panels and 49 time periods, Pm was the most appropriate. Based on the Fisher-type panel unit root test results, all p-values are less than 5% for all the variables included in the model (except for mangrove cover, total population, Per Capita Net State Domestic Product, Capital Expenditure and Revenue Expenditure). Therefore, the first difference was taken to convert these variables from non-stationary to stationary. After that, all the variables P values are significant at the 5% level; hence, we rejected the null hypothesis that all panels contain unit roots, which is generally sufficient to proceed with panel data regression.

#### Multicollinearity checks

A VIF > 5 (or 1/VIF < 0.2) suggests potential multicollinearity, while VIF > 10 (or 1/VIF < 0.1) indicates serious multicollinearity that may distort regression results. Hence, we checked for multicollinearity for the variables included in the model. Except for the variables revenue expenditure, per capita net state domestic product, and capital expenditure, all variables had multicollinearity issues. To avoid this, group mean centering (also known as within-group centering or demeaning) was performed to remove the multicollinearity issue.

#### Test for endogeneity, instrument strength, overidentifying restrictions

Endogeneity was tested using the IV (2SLS) regression (Table 3). The model was statistically significant overall (F(6,12) = 187.92, p < 0.001), with a high centered R^2^ of 0.96, indicating that the instruments explained a large portion of the variation in the dependent variable. Among the regressors, ‘population affected’ and ‘damage to public utilities’ were highly significant (p < 0.001), suggesting strong positive effects on the dependent variable. The under-identification test (Kleibergen-Paap LM) has a p-value of 0.40, suggesting that the model is not under-identified. In addition, the weak identification test (Cragg-Donald F = 0.04) indicates potential strong instruments. The Hansen J test (p = 0.59) shows that the instruments are valid and do not over-identify the model. Finally, the endogeneity test (p = 0.04) rejects the null hypothesis, suggesting that the model does not have an endogeneity issue.

**Table 3:** Test for Endogeneity.

			Number of obs	=	19	
			F( 6, 12)	=	187.92	
			Prob > F	=	0.00	
Total (centered) SS = 285,086,574.5			Centered R^2^	=	0.96	
Total (uncentered) SS = 322,490,099.6			Uncentered R^2^	=	0.97	
Residual SS = 10,436,164.95			Root MSE	=	741.10	
		Robust				
$TD$	Coefficient	Std. err	Z	P > z	[95% conf. interval]	
$CE$	− 0.072^NS^	0.155	− 0.470	0.641	− 0.376	0.232
$RE$	0.018^NS^	0.078	0.230	0.821	− 0.135	0.170
$GENC$	− 0.004^NS^	0.007	-0.600	0.549	− 0.019	0.010
$PA$	510.551***	106.212	4.810	0.000	302.378	718.723
$DTH$	− 0.003^NS^	0.003	− 1.010	0.311	− 0.008	0.003
$DTPU$	1.298***	0.068	18.950	0.000	1.164	1.432
$MC$	− 2.64	74.78	− 0.04	0.97	− 149.19	143.92
_cons	250.312^NS^	621.600	0.400	0.687	− 968.002	1468.626
	Under-identification test (Kleibergen-Paap rk LM statistic): 4.070 Chi-sq(4) P-val = 0.3966^NS^					
	Weak identification test (Cragg-Donald Wald F statistic): 0.038** (Kleibergen-Paap rk Wald F statistic): 2.243					
	Hansen J statistic (overidentification test of all instruments): 1.960 Chi-sq(3) P-val = 0.5808^NS^ -endog- option: Endogeneity test of endogenous regressors: 10.018 Chi-sq(6) P-val = 0.0382** Regressors tested: CE, RE, GENC, PA, DTH, DTPU, MC					
	Instrumented: : CE, RE, GENC, PA, DTH, DTPU, MC Excluded instruments: ARF, PCNSDP, FA, TP, AA, DTCA, CL, HLL					

*** indicates 1% significance, ** indicates 5% significance, and NS indicates Not Significant.

#### Estimation of FE and RE models

If endogeneity exists, then we have to use IV/2SLS/GMM; however, the endogeneity results showed that there were no such issues. Hence, to assess the determinants of flood vulnerability, fixed and random effect models were used. Since our endogeneity test showed no significant endogeneity, the variables previously treated as endogenous in the IV model can now be treated as exogenous. Therefore, in our Fixed Effects (FE) and Random Effects (RE) models, we excluded the instrument variables (eight instrument variables) because they were used solely to address endogeneity in the IV model and were not explanatory variables in the structural equation. After executing the FE and RE models, the Hausman test was performed. The Hausman test compares the Fixed Effects (FE) and Random Effects (RE) models to determine whether the RE model’s assumption that individual effects are uncorrelated with the regressors is valid. In our results, the test statistic is χ^2^(3) = 2.03 with a p-value of 0.57, which is well above the conventional 0.05 threshold. This means that we fail to reject the null hypothesis that the difference in coefficients is not systematic. Therefore, the RE model is preferred, as it is more efficient under the null and the assumption of no correlation between individual effects and regressors (Table 4).

**Table 4:** Hausman test.

	Coefficients			
	(b)	(B)	(b-B)	sqrt(diag(V_b-V_B))
	fe	Re	Difference	Std. err
$CE$	− 0.005	− 0.008	0.003	0.055
$RE$	− 0.043	− 0.039	− 0.004	0.063
$GENC$	− 0.002	− 0.002	0	0.000
$PA$	277.047	261.899	15.148	3.892
$DTH$	0.003	0.002	0.001	0.032
$DTPU$	1.039	1.093	− 0.054	0.232
$MC$	− 42.105	− 44.229	2.124	1.457
	b = Consistent under H0 and Ha; obtained from xtreg			
	B = Inconsistent under Ha, efficient under H0; obtained from xtreg			
	Test of H_0_: Difference in coefficients not systematic			
	chi^2^(3) = (b-B)'[(V_b-V_B)^(-1)](b-B)			
	2.03			
	Prob > chi^2^ = 0.5653			

#### Diagnostics and robustness checks

Subsequently, we conducted diagnostic and robust checks. It began with checking for autocorrelation in the panel data (Table 5). The Wooldridge test for autocorrelation in panel data yields an F-statistic of 8.304, with a p-value of 0.009, which is statistically significant at the 1% level. This means that we reject the null hypothesis of no first-order autocorrelation, indicating that autocorrelation is present in the panel data. This suggests that the standard errors may be biased; hence, we considered using robust or clustered standard errors to correct for this issue.

**Table 5:** Random effect results with cluster-robust standard errors.

Random-effects GLS regression				Number of obs = 224		
Group variable: state_n				Number of groups = 26		
				Obs per group:		
R-squared:				Min = 1		
Within = 0.7336				Avg = 8.6		
Between = 0.92				Max = 12		
Overall = 0.7361						
				Wald chi^2^(6) = 493.2		
corr(u_i, X) = 0 (assumed)				Prob > chi^2^ = 0.0000		
			(Std. err. adjusted for 26 clusters in state_n)			

centered_td	Coefficient	Robust std.err	z	P > z	[95% conf. interval]	
$CE$	− 0.008**	0.004	− 2.276	0.023	− 0.015	− 0.001
$RE$	− 0.039^NS^	0.035	− 1.114	0.265	− 3.074	0.846
$GENC$	− 0.002***	0.001	− 3.328	0.001	− 0.003	− 0.001
$PA$	261.899***	43.993	5.953	0.000	175.674	348.124
$DTH$	0.002**	0.001	2.000	0.046	0.000	0.004
$DTPU$	1.093***	0.078	14.013	0.000	0.940	1.246
$MC$	− 44.229***	16.617	− 2.662	0.008	− 76.798	− 11.660
constant	213.378^NS^	260.001	0.820	0.412	− 296.215	722.971
sigma_u	933.683					
sigma_e	1673.525					
Rho	0.237	(fraction of variance due to u_i)				

*** indicates 1% significance, ** indicates 5% significance, and NS indicates Not Significant.

The random-effects GLS regression model examines the relationship between the dependent variable (total damage due to flood, TD) and various independent variables across 26 states using 224 observations. The results indicate that the independent variables collectively explain a significant portion of the variance, with an overall R-squared value of 0.736. The Wald chi-squared test statistic of 493.20 and p-value of 0.0000, less than one, confirm the model’s statistical significance, suggesting that the included independent variables meaningfully influence the dependent variable.

Several factors emerged as statistically significant predictors of damage. Capital expenditure (CE) has a negative and significant coefficient (− 0.008, p = 0.023), suggesting that increased capital investment contributes to reducing flood damage. Government Expenditure on Natural Calamities (GENC) also shows a negative relationship, with a highly significant coefficient (− 0.002, p = 0.001), reinforcing the idea that increased public spending lowers the extent of damage. Notably, mangrove coverage (MC) had a strong protective effect, with a coefficient of − 44.229 (p = 0.008), highlighting the role of natural barriers in mitigating disaster-related damage.

Mangrove forests play a crucial role in mitigating the impacts of climate change and reducing damage from natural disasters. They act as natural barriers to coastal abrasion and provide protection against extreme weather events^126,127^. The economic benefits of mangrove forests, including protection from climate-related damage, exceed the investment costs of rehabilitation projects^126^. Government expenditure, particularly in the form of public investment, can significantly impact economic growth and, by extension, a country’s ability to respond to natural calamities. The optimal composition of public investment and consumption in government expenditure can lead to stronger growth effects^128^. This increased economic capacity could potentially contribute to improved disaster preparedness and response. Mangrove conservation and restoration efforts, which may be reflected in government expenditure, can help maintain the ecosystem services provided by these forests. These services include coastal protection and carbon sequestration, which indirectly contribute to reducing damage from natural disasters^129–131^.

Conversely, the other variables showed a positive association with the damages. The population affected by floods (PA) has a large and highly significant coefficient (261.899, p = 0.000), indicating that more affected people correspond to greater damage levels. Damage to houses (DTH) also had a positive and significant impact (coefficient: 0.002, p = 0.046), suggesting that health-related consequences escalate alongside total damage. The most pronounced positive effect comes from damage to public utilities due to floods (DTPU), which has a coefficient of 1.093 (p = 0.000), implying that infrastructure losses contribute heavily to damage estimates.

The number of people affected and the damage to houses are significant factors in flood damage costs. Kok et al.^105^ reported that in the 2021 Netherlands floods, more than 2,500 houses and 5,000 inhabitants were affected, contributing to a total damage estimate of €350–600 million. Physical damage to houses and businesses is among the most substantial components of total damage^105^. Damage to public utilities, although not explicitly quantified in most studies, is implied to be a significant factor. They also mentioned that damage to infrastructure is one of the most substantial components of total damage costs. Tanoue et al.^20^ discussed how floods can cause direct economic damage through the destruction of physical assets, including public utilities. The relationship between these variables and the total damage costs is complex and context-dependent. Vogel et al.^132^ suggested that flood loss is influenced by multiple factors, including hydrological and hydraulic aspects, building characteristics, and property-level mitigation measures. This indicates that the impact of the population affected, house damage, and public utility damage on the total costs may vary depending on local conditions and flood types.

Overall, these findings suggest that public investment, particularly in capital expenditure and mangrove conservation, significantly reduces damage. Population exposure and infrastructure losses remain key drivers of increased damage, highlighting the need for targeted interventions to strengthen resilience in vulnerable areas. Policymakers should consider strategies that focus on infrastructure protection and disaster preparedness to effectively mitigate future risks.

## Conclusion and policy implications

This study provides a comprehensive analysis of the socioeconomic and environmental determinants influencing flood vulnerability across Indian states using a robust panel-dataset approach. The findings underscore the critical roles of public investment and environmental conservation in mitigating flood-related damage. The Random Effects model, validated through the Hausman test, reveals that capital expenditure, government spending on natural calamities, and mangrove coverage significantly reduce flood damage. These results highlight the effectiveness of proactive fiscal and ecological strategies in enhancing flood resilience.

Conversely, variables such as the number of people affected, damage to houses, and damage to public utilities were positively associated with total flood damage, indicating that population exposure and infrastructure vulnerability are key drivers of disaster impact. The presence of autocorrelation in the panel data, addressed through cluster-robust standard errors, further strengthens the reliability of the model estimates.

This study emphasizes the importance of integrating environmental protection, particularly mangrove conservation, with strategic public investment to reduce disaster vulnerability. This also highlights the need for targeted interventions in densely populated and infrastructure-sensitive regions. Overall, this research provides actionable insights for policymakers aiming to build a more resilient and adaptive disaster management framework in India.

Based on the analysis of flood impacts in India, the policy implications are as follows.

*Increase Capital Expenditure on Flood-Resilient Infrastructure:* Allocate greater public investment toward building and upgrading flood-resistant infrastructure, such as embankments, drainage systems, and resilient housing, especially in flood-prone regions.

*Enhance Government Spending on Disaster Preparedness:* Strengthen budgetary allocations for natural calamities, focusing on early warning systems, emergency response mechanisms, and community-based disaster risk reduction programs.

*Promote Mangrove Conservation and Restoration:* Implement large-scale mangrove afforestation and conservation projects, particularly in coastal states, to leverage their natural flood-buffering capacity and ecosystem services.

*Develop Targeted Support for High-Risk Populations:* Identify and prioritize interventions in areas with high population exposure to floods. This includes relocation assistance, flood insurance schemes, and resilient housing for vulnerable communities in the Philippines.

*Protect and Upgrade Critical Public Utilities:* Invest in the protection and climate-proofing of essential public utilities, such as water supply, electricity, and transportation networks, to minimize service disruption and economic losses during floods.

## Supplementary Information

Supplementary Information.

## Data Availability

The datasets used and/or analysed during the current study are attached as Supplementary files.
